# Drought Stress, Elevated CO_2_ and Their Combination Differentially Affect Carbon and Nitrogen in Different Organs of Six Spring Wheat Genotypes

**DOI:** 10.3390/plants13202942

**Published:** 2024-10-21

**Authors:** Sajid Shokat, Fulai Liu, Dominik K. Großkinsky

**Affiliations:** 1 Section for Crop Science, Department of Plant and Environmental Sciences, University of Copenhagen, Højbakkegård Allé 13, 2630 Taastrup, Denmark; fl@plen.ku.dk; 2 Wheat Breeding Group, Plant Breeding and Genetics Division, Nuclear Institute for Agriculture and Biology, Faisalabad 38000, Pakistan; 3 Center for Health and Bioresources, Bioresources Unit, AIT Austrian Institute of Technology GmbH, Konrad-Lorenz-Straße 24, 3430 Tulln, Austria

**Keywords:** bread wheat, carbon dioxide, climate change, CN ratio, drought, field crop, food security, landrace derivatives, stress tolerance, synthetic derivatives

## Abstract

This study aimed to analyze the combined impact of CO_2_ and drought stress at the flowering stage on carbon (C), nitrogen (N), and CN ratios in leaves, stem, and grains of bread wheat. Six diverse bread wheat genotypes, comprised of two commercial checks, two landraces, and two synthetics derivatives, were grown at two levels of CO_2_, i.e., 400 ppm and 800 ppm, and drought stress was imposed at the flowering stage through progressive soil drying. Stem, leaf, and grain samples were taken at maturity and concentrations of C and N were determined. Our results indicate that the threshold value of fraction of transpirable soil water (C_FTSW_) at which it diverges towards closure of stomata was different among genotypes and a higher range of values was estimated under elevated CO_2_. Drought significantly increased C levels in leaves and N levels in grains but decreased N levels in leaves, which increased CN ratios in leaves. In contrast, drought significantly reduced CN ratios in grains. Genotypes differed significantly in N content in grains, where the landrace derivative L_2_ maintained the highest N content. Moreover, pronounced changes in leaf N and CN ratios were induced by the combination of elevated CO_2_ and drought stress. Additionally, combined correlation and biplot analyses indicate a strong positive association of grain CN (GCN) with grain number, weight, and grain yield. These effects possibly interact with drought to strongly interfere with the impact of elevated CO_2_. The differential performance of the tested genotypes shows that selection of appropriate germplasm is essential to maintain agricultural production.

## 1. Introduction

Climate change is an alarming threat to sustainable agricultural production and food security. This ever-increasing menace has forced scientists to explore the performance of plants under respective conditions. Recent studies have explored the impact of drought and elevated CO_2_ on different crop species including wheat [1,2]. Specifically, reports suggest that the severity of drought effects can be alleviated by elevated CO_2_ [3,4], while other indicates that elevated CO_2_ cannot improve plant performance under drought conditions [5]. Therefore, no conclusive prediction can be made about the impact of elevated CO_2_ on drought tolerance, as it can vary even within the species [5].

Progressive soil drying is considered a potential tool for the precise evaluation of plant germplasm under drought stress, and determination of the fraction of transpirable soil water (FTSW), along with its threshold (C_FTSW_), can indicate the plant functions towards closure of stomata in wheat, barley, tomato, and soybean [5,6]. Hence, estimation of C_FTSW_ can be used to explain plant performance under drought conditions.

Likewise, intensive studies have been conducted to understand the interactive effect of drought on plant responses to physiology and carbon assimilation and its allocation [7,8]. Studies on wheat have indicated that elevated CO_2_ improves the plant’s physiological responses by improving its water use efficiency, antioxidant potential, and ultimately the grain yield under abiotic stresses [4,9]. Further, another study indicates that elevated CO_2_ does not improve grain yield and that many physiological responses are suppressed [5]. This study also indicates that cross-talk among phytohormones, especially cytokinins (trans-zeatin), and ratio of salicylic acid to abscisic acid are important for wheat adaptation under the combined effect of drought and elevated CO_2_ [5]. However, most of these investigations focused on one to two genotypes and more comprehensive and systematic studies are needed to conclude anything.

Many studies of elemental composition have indicated that elevated CO_2_ enhances carbon assimilation, probably due to the higher photosynthetic rate in wheat and rice [8,10]. Likewise, translocation of nutrients is also well documented in different plant tissues [10]. However, limited studies have been conducted on a set of diverse germplasm originated from landraces, synthetic, and elite cultivars of bread wheat to understand the combined impact of drought and elevated CO_2_ in different tissues of bread wheat. Studies have shown that plant genetic diversity and its interaction with different climate factors is an important aspect in predicting plant performance [11,12]. Landraces and synthetics are considered to be hubs of plant genetic diversity and bread wheat has been improved against certain biotic and abiotic stresses, exploiting derivatives of landraces and synthetic bread wheat [13,14]. Stress effects on certain plant traits, including grain number and weight, have been studied in these exotic resources and a few novel genes for drought and heat tolerances have been derived from them [10,14]. As CN ratios have not been studied in this germplasm so far, the potential of this trait, especially in response to environmental conditions, was targeted. The current study is designed with the hypothesis that genetic diversity originating from landraces and synthetic bread wheat has the potential to sustain carbon, nitrogen, and CN ratio under drought conditions and elevated CO_2_. Six diverse genotypes were used in this study, of which two originated from landraces, two from synthetic bread wheat, and two from commercial cultivars, to test our hypothesis by quantifying the impact of drought and elevated CO_2_ on carbon and nitrogen in leaves, stem, and grains.

## 2. Results

### 2.1. Threshold Value of Fraction of Transpirable Soil Water (FTSW)

The fraction of transpirable soil water (FTSW) was determined by measuring the daily water consumption of each pot. Values of FTSW were drawn against daily evapotranspiration to drive the threshold values of FTSW (C_FTSW_). Under ambient CO_2_, the highest C_FTSW_ values (0.61) were recorded for genotypes L_1_ and L_4_, while L_3_ exhibited the lowest C_FTSW_ value (0.39), and the genotypes L_2_, L_5_, and L_6_ showed intermediate C_FTSW_ values of 0.46, 0.55, and 0.43, respectively. In contrast, the highest C_FTSW_ values (0.72) were monitored for genotypes L_3_ and L_5_ under elevated CO_2_, while L_6_ showed an intermediate C_FTSW_ value of 0.64 and the other three genotypes showed low C_FTSW_ values between 0.53 and 0.55 (Table S2).

### 2.2. Carbon, Nitrogen, and CN Ratios in Stem, Leaves, and Grains

Drought stress at the flowering stage significantly affected leaf carbon (LC), nitrogen (LN), grain nitrogen (GN), and the carbon to nitrogen (CN) ratio of stem (SCN), leaf (LCN), and grains (GCN) in comparison to well-watered conditions. LC and GN increased, and GCN decreased under drought conditions (Table 1). However, non-significant effects of drought were observed for stem carbon (SC) and grain carbon (GC). Elevated CO_2_ had a significant impact on SC, LN, GC, GN, and GCN; however, no general trend was observed under elevated CO_2_. Interestingly, effects of elevated CO_2_ were non-significant in improving LC, SN, LCN, and SCN. Significant differences were observed among genotypes for the three traits, i.e., LC, GN, and GCN. The highest LC and GN were determined in genotype L_2_ and the highest GCN in L_6_. In contrast, these genotypes differed non-significantly for SC, SN, LN, GC, SCN, and LCN. The interaction between the levels of water and CO_2_ (Pw × CO_2_) was significant for LN and LCN, and a pronounced change in these parameters was recorded under the combined effect of drought and elevated CO_2_. It is further suggesting that LN and LCN can be affected severely under conditions of low water and high CO_2_. Moreover, interaction between genotypes and water levels (Pw × G) was also significant for SN, where a decreasing trend was observed in five genotypes and a maximum reduction for SN was recorded in genotype L_2_ under drought conditions. These results exhibit that flowering-stage drought stress does not enhance the SN in the used set of germplasm.

### 2.3. Combined Correlation and Principal Component Analysis

**Figure 1:**
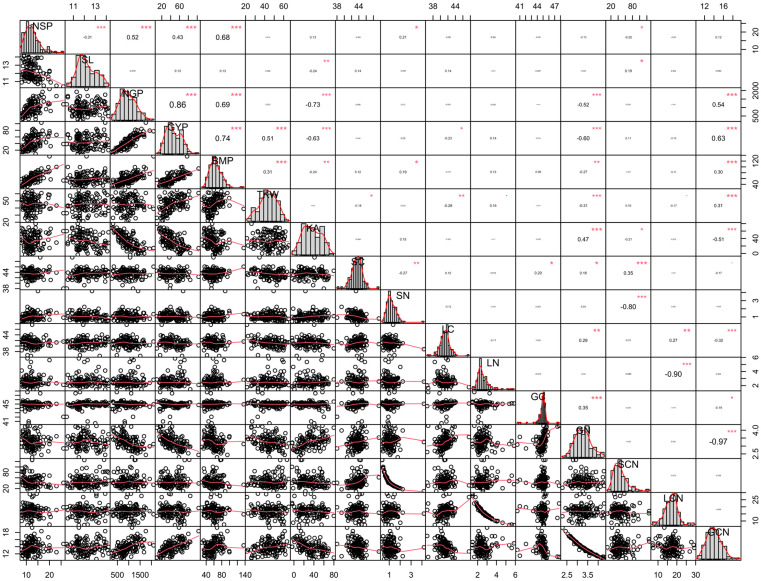
Combined correlation analysis indicates the relationship of carbon (C) and nitrogen (N) elements with grain yield and important yield traits of six bread wheat genotypes grown under ambient (400 ppm) and elevated CO_2_ (800 ppm) concentrations exposed to well-watered (WW) and drought stress (D) conditions. NSP is number of spikes^−1^, SL is spike length (cm), NGP is number of grain plants^−1^, GYP is grain yield pot^−1^ (g), BMP is biomass pot^−1^ (g), TKW is thousand kernel weight (g), SC is stem carbon (mg g^−1^), SN is stem nitrogen (mg g^−1^), LC is leaf carbon (mg g^−1^), LN is leaf nitrogen (mg g^−1^), GC is grain carbon (mg g^−1^), GN is grain nitrogen (mg g^−1^), SCN is stem carbon to nitrogen ratio, LCN is leaf carbon to nitrogen ratio, and GCN is grain carbon to nitrogen ratio. Further, “*” indicates the significance at 0.05% probability, “**” shows the significance at 0.01% probability, and “***” exhibits the significance at 0.001% probability.

A combined correlation of the six genotypes grown at two levels of CO_2_ and water indicates that the number of grain spikes^−1^, grain yield, biomass, and thousand kernel weight were significantly and positively correlated with GCN. It also indicates that higher GCN is probably attained by maintaining higher biomass and grain weight. In contrast, GN was significantly and negatively correlated with the aforementioned traits. No significant correlation of the above-mentioned yield-related traits with SCN and LCN was found (Figure 1).

**Figure 2:**
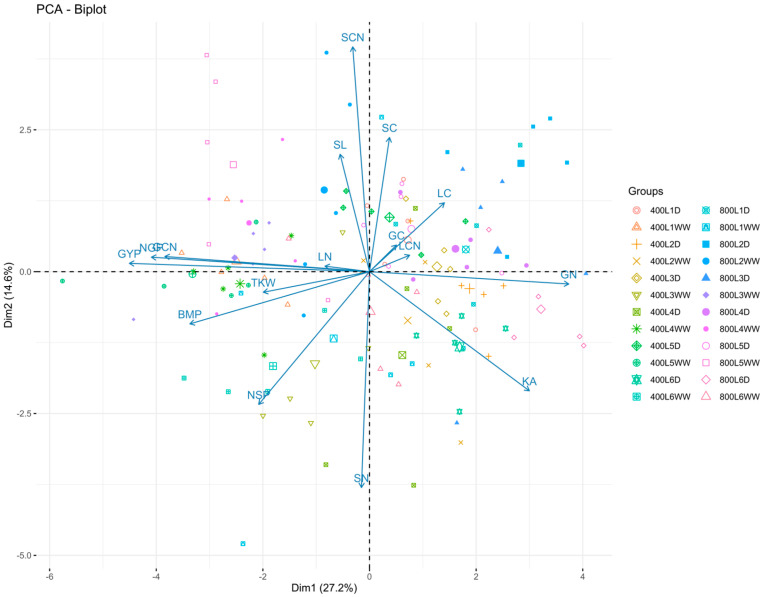
Biplot of combined principal component analysis is indicating the relationship of carbon (C) and nitrogen (N) elements with grain yield and important yield traits of six bread wheat genotypes grown under ambient (400 ppm) and elevated CO_2_ (800 ppm) concentrations exposed to well-watered (WW) and drought stress (D) conditions. NSP is number of spikes^−1^, SL is spike length (cm), NGP is number of grain plants^−1^, GYP is grain yield pot^−1^ (g), BMP is biomass pot^−1^ (g), TKW is thousand kernel weight (g), SC is stem carbon (mg g^−1^), SN is stem nitrogen (mg g^−1^), LC is leaf carbon (mg g^−1^), LN is leaf nitrogen (mg g^−1^), GC is grain carbon (mg g^−1^), GN is grain nitrogen (mg g^−1^), SCN is stem carbon to nitrogen ratio, and LCN is leaf carbon to nitrogen ratio.

A combined principal component analysis of six genotypes grown at two levels of CO_2_ and water explains 27.2% and 14.6% of the variability for PC_1_ (Dim1) and PC_2_ (Dim2), respectively. A biplot was drawn between these two PCs to indicate the direction of the relationship between GCN and yield-related traits. Again, grain yield, number of grain spikes^−1^, biomass, thousand kernel weight, and GCN were located in the same direction but in the opposite direction to GN (Figure 2). Further, no significant associations of these yield-related traits were recorded with SCN and LCN, indicating that GCN can be a reliable trait to indicate the better performance of wheat under drought and elevated CO_2_ (Figure 2).

## 3. Discussion

Evaluation of bread wheat under precise drought conditions is one of the major objectives of drought experiments. Irrigating the plants with a defined quantity of water and estimating daily evapotranspiration can provide a fair evaluation of germplasm [5,6]. Additionally, the fraction of transpirable soil water (FTSW) indicates the volumetric soil moisture content at a given time in relation to the total amount of transpirable soil water. In this study, values of FTSW were drawn against evapotranspiration to predict the threshold value (C_FTSW_). Previous studies have indicated that C_FTSW_ is lower under elevated CO_2_ in comparison to ambient CO_2_ [15]; however, the results of the current experiment cannot identify any general trend. Interestingly, values of C_FTSW_ were higher under elevated CO_2_, indicating that wheat genotypes grown under these conditions tend towards closure of stomata earlier than when grown under ambient levels of CO_2_.

Carbon to nitrogen ratio (CN) is one of the indicators to express the metabolic and especially the nutrient status of plants [16], which changes under water-limiting conditions. An analysis of different plant tissues derived from diverse wheat genotypes can reveal the adaptation strategies of plants to the combined impacts of drought and elevated CO_2_. Drought stress has been reported to change photosynthesis, carbon, and nitrogen mainly due to early senescence in plants [17,18,19]. Our earlier studies on the same germplasm reported a reduction in photosynthesis [5]; here, we also recorded a change in LC and CN in leaves, stem, and grains under drought conditions, where generally LCN and GCN were decreased. Our results further indicating that drought affects the grain nutrient status through a change in LCN; however, more in-depth studies are required. Meta-analysis of important studies at a global level has indicated that higher CO_2_ levels can promote plant productivity as well as leaf CN ratio [20]. In contrast, our results indicated an increasing trend of LN and resultantly a decreasing trend of LCN under elevated CO_2_. Another study conducted on wheat at multiple sites in Europe with different levels of nitrogen indicated that GN decreased by 15% when CO_2_ was increased from 360 ppm to 680 ppm [21]. In contrast, we recorded an increasing trend in GN and ultimately a decreasing trend in GCN under elevated CO_2_ and under the combined effect of drought and elevated CO_2_. We speculate that the used set of germplasm probably maintained better GN due to better translocation of nitrogen from leaves to sink, which helped the plants to maintain lower GCN under combined drought and elevated CO_2_.

We also studied the effect of elevated CO_2_ on the elemental status and CN ratio of leaf, stem, and grains. Our results indicated a higher CN ratio in stem in comparison to leaf and grain. It has been reported that drought stress decreases CN while elevated CO_2_ ameliorates the negative impact on CN ratio [22]. In line with this, we found a decrease in CN under drought stress, while it was increased under elevated CO_2_ both in stem and leaf. Wang et al. [22] reported that the combined effect of drought and elevated CO_2_ helps to maintain the CN ratio, with higher activity of invertase and catalase, which further stimulates plant root exudation. We found that genotype L_6_ and L_5_ had the highest GCN, while genotype L_2_ showed the lowest. Earlier studies on winter wheat have indicated that SCN has no association with wheat performance [23] and we also find no association of this trait with grain yield. However, our analysis through combined correlation and PCA indicates that GCN has a strong positive association with biomass, thousand grain weight, and grain yield, explaining that wheat probably maintained better GCN due to improved biomass and grain weight. It also indicates that wheat genotypes that maintained higher GCN also produced a higher grain yield. Previous studies have shown that bread wheat of synthetic origin has the potential to maintain optimum grain yield under drought stress [5,12]. Our results also indicate that wheat germplasm that originated from synthetic bread wheat has the potential to maintain a positive association of grain yield with GCN (Table S1; Figure 1 and Figure 2). Further, the same genotypes also maintained lower C_FTSW_, indicating that genotypes probably maintained better GCN due to late closure of stomata under drought conditions (Table S2). In contrast, no increase in GCN was recorded under the combined effects of elevated CO_2_ and drought stress, indicating that higher CO_2_ does not improve the metabolic or nutrient status in the used set of genotypes.

## 4. Materials and Methods

### 4.1. Plant Material

Six spring wheat genotypes exhibiting contrasting levels of drought tolerance under field conditions were used to conduct the experiment. Five of these genotypes (L_1_, L_2_, L_3_, L_4_, and L_5_) were developed at the International Maize and Wheat Improvement Centre (CIMMYT), Mexico, and one genotype (L_6_) was developed at Ayub Agricultural Research Institute (AARI), Pakistan. Field data indicated that genotypes L_1_ and L_2_ had better drought tolerance, L_3_ and L_4_ were moderately drought tolerant, while L_5_ was selected as a drought-tolerant check and L_6_ was a drought-sensitive check (Table S1). Plastic pots with a capacity of 4 L were selected and filled with soil comprising peat material, Sphagnum, 32% organic matter, pH = 5.6–6.4 and EC = 0.45 mS cm^−1^. Four seeds were sown in each pot and, after one week of emergence, thinning was carried out to leave two seedlings only.

### 4.2. Growth Conditions

Two sets of genotypes were grown in two completely controlled greenhouse cells. In cell-1, CO_2_ was maintained at 400 ppm along with other environmental conditions. Likewise, in cell-2, all conditions were the same, except CO_2_ which was kept at 800 ppm throughout the growth period. The size of each greenhouse cell was 50 m^2^ and the temperature was maintained at 22/16 °C day/night. The light period was 16/8 h day/night [photosynthetic active radiation was 360 μmol m^−2^ s^−1^ and 0.5 μmol m s^−1^ for day and night, respectively]. The range of relative air humidity was 55–60%. Details about the maintenance of CO_2_ have been described previously [4,5]. All six genotypes were grown with 1.8 kg peat material after determining their maximum water holding capacity. After a few days of emergence, automatic fertigation (irrigation + mixture of essential nutrients) was applied to the plants. Furthermore, the weight of each pot was kept at the same water level by manual weighing of the pots.

Maximum water-holding capacity was maintained until flowering under all conditions. At flowering time, 4 replications of each genotype were harvested to study different agro-physiological parameters before applying drought stress. The remaining 20 replications of each genotype were divided into two sets: irrigation was withdrawn during anthesis for one set (10 pots) and water status of the other set (10 pots) was kept at 95% water holding capacity of pot. Daily evapotranspiration (ET) of each pot was recorded by weighing. Total transpirable soil water was the change in the pot weight between 95% water holding capacity (about 3.2 kg pot weight) and when evapotranspiration of the drought plants decreased to 10% of the well-watered plants (when pot weight was ca. 1.6 kg), and the soil water status in the pot was expressed as the fraction of transpirable soil water (FTSW).

FTSW = WTn − WTf/TTSW

where WTn is the actual pot weight on a given date and WTf is the pot weight at the time when transpiration rate of stressed plants was 10% of the well-watered plants (pot weight about 3.1 kg). TTSW is the total transpirable soil water.

### 4.3. Sampling and Measurement of Carbon and Nitrogen of Leaves, Stems, and Grains

Samples of leaves, stems, and grains were taken at maturity. Samples of leaves and stems were dried at 65 °C for 72 h. A Cyclone mill twister (Retsch) was used for grinding the samples and a CHNS/O Elemental Analyser (Flash 2000, Thermo Fisher Scientific, Cambridge, UK) was used to determine carbon (C) and nitrogen (N) content. Calculation of C and N of these samples was estimated by determining the N content (g) of leaves, stems, and grains and then by multiplying with the biomass of the organ, respectively.

### 4.4. Statistical Analysis

A linear-plateau model was used to draw the responses of daily evapotranspiration to progressive soil drying. Means and standard errors were calculated in Microsoft Excel 2010.Ink using respective formulae. To test our null hypothesis, a three-way analysis of variance (ANOVA) was conducted to differentiate genotypes, CO_2_ levels, water levels, and their interactions. Further, an F-test was used to identify significant differences based on *p*-values derived from the F table. Likewise, correlation matrixes and principal component analyses were prepared in RStudio 1.0.153. The package performance analytics was used to draw the correlation, and packages devtools, factoextra, and fviz_pca_biplot were used to draw principal component analysis and biplots.

## 5. Conclusions

In conclusion, C_FTSW_ can show the differences among diverse wheat genotypes and this germplasm also differs in GCN in response to drought. Although elevated CO_2_ can improve plant gas exchange parameters, it did not improve grain yield or GCN in the current set of germplasm. Our results also support the observation that selection of a suitable genotype is very important to attain optimum grain yield and CN, also under adverse conditions. Hence, GCN can serve as a pre-breeding trait to attain optimum grain yield.

## Figures and Tables

**Table 1:** Means and standard errors for the impact of ambient and elevated CO_2_ on six genotypes for carbon and nitrogen amount and their ratios in leaf, stem, and grain grown under well-watered and drought conditions.

Genotypes	CO_2_ Level	Conditions	SC (mg g^−1^)	SN (mg g^−1^)	LC (mg g^−1^)	LN (mg g^−1^)	GC (mg g^−1^)	GN (mg g^−1^)	SCN (%)	LCN (%)	GCN (%)
L_1_	400	WW	43.55 ± 0.22	1.16 ± 0.07	39.63 ± 0.82	2.32 ± 0.09	44.87 ± 0.11	2.78 ± 0.08	38.26 ± 2.51	17.12 ± 0.45	16.17 ± 0.44
		D	43.39 ± 0.3	0.98 ± 0.06	41.12 ± 0.36	2.38 ± 0.06	43.96 ± 0.85	3.23 ± 0.09	45.1 ± 2.75	17.3 ± 0.52	13.63 ± 0.36
	800	WW	43.62 ± 0.19	1.89 ± 0.61	40.82 ± 0.55	3.1 ± 0.27	45.15 ± 0.06	3.28 ± 0.03	35.18 ± 12.36	13.49 ± 0.92	13.76 ± 0.12
		D	42.63 ± 0.58	1.06 ± 0.2	41.87 ± 0.25	2.78 ± 0.26	45.02 ± 0.14	3.47 ± 0.1	46.67 ± 9.39	15.69 ± 1.63	13 ± 0.35
L_2_	400	WW	42.42 ± 0.86	1.41 ± 0.27	41.09 ± 0.67	2.52 ± 0.21	44.80 ± 0.15	3.63 ± 0.12	34.9 ± 6.35	16.71 ± 1.24	12.39 ± 0.37
		D	42.97 ± 0.25	1.09 ± 0.13	42.15 ± 0.33	2.33 ± 0.1	45.1 ± 0.11	3.83 ± 0.12	41.34 ± 4.28	18.19 ± 0.78	11.83 ± 0.39
	800	WW	43.26 ± 0.59	0.85 ± 0.22	42.64 ± 0.27	2.38 ± 0.22	44.91 ± 0.04	3.36 ± 0.07	64.03 ± 13.65	18.55 ± 1.68	13.4 ± 0.26
		D	45.34 ± 0.37	0.78 ± 0.15	42.5 ± 0.45	2.79 ± 0.15	45.29 ± 0.08	3.91 ± 0.15	64.99 ± 9.23	15.42 ± 0.9	11.65 ± 0.46
L_3_	400	WW	43.22 ± 0.45	1.43 ± 0.19	40.84 ± 0.31	2.14 ± 0.07	45.03 ± 0.08	3.32 ± 0.11	33.25 ± 5.9	19.2 ± 0.54	13.61 ± 0.41
		D	43.61 ± 0.18	1.02 ± 0.08	41.31 ± 0.56	2.25 ± 0.07	44.9 ± 0.09	3.24 ± 0.14	43.84 ± 3.77	18.44 ± 0.61	13.95 ± 0.57
	800	WW	45.22 ± 0.33	0.9 ± 0.12	40.27 ± 0.67	4.37 ± 0.51	44.93 ± 0.08	3.13 ± 0.05	53.06 ± 5.8	9.82 ± 1.35	14.38 ± 0.21
		D	43.6 ± 0.63	0.99 ± 0.24	40.88 ± 0.71	2.29 ± 0.15	45.21 ± 0.18	3.58 ± 0.11	54.38 ± 11.87	18.27 ± 1.53	12.66 ± 0.33
L_4_	400	WW	42.11 ± 0.4	0.86 ± 0.07	40.3 ± 0.45	2.98 ± 0.29	44.53 ± 0.19	2.92 ± 0.09	50.32 ± 4.57	14.02 ± 1.28	15.33 ± 0.49
		D	42.61 ± 1.88	1.03 ± 0.2	40.31 ± 0.38	2.83 ± 0.21	44.69 ± 0.86	3.50 ± 0.22	47.37 ± 8.01	14.68 ± 1.39	12.91 ± 0.56
	800	WW	44.44 ± 0.5	1.06 ± 0.26	40.86 ± 0.2	3.49 ± 0.77	45.06 ± 0.04	2.86 ± 0.05	52.24 ± 11.05	14.08 ± 2.78	15.75 ± 0.25
		D	43.99 ± 0.4	1.06 ± 0.14	43.05 ± 1.26	2.23 ± 0.19	45.09 ± 0.07	3.37 ± 0.08	44.96 ± 6.85	19.91 ± 2	13.42 ± 0.29
L_5_	400	WW	43.09 ± 0.38	1.07 ± 0.11	40.64 ± 0.12	2.16 ± 0.07	44.85 ± 0.05	2.75 ± 0.12	42.98 ± 6.14	18.91 ± 0.58	16.46 ± 0.72
		D	43.64 ± 0.23	0.85 ± 0.02	41.68 ± 0.2	2.44 ± 0.09	44.83 ± 0.19	3.11 ± 0.15	51.33 ± 1.77	17.21 ± 0.7	14.52 ± 0.58
	800	WW	42.56 ± 0.76	0.61 ± 0.14	40.32 ± 0.46	2.57 ± 0.33	45.22 ± 0.08	2.89 ± 0.07	87.05 ± 18.66	17.08 ± 2.82	15.68 ± 0.4
		D	44.68 ± 0.12	1.19 ± 0.13	42.22 ± 0.5	1.96 ± 0.21	45.42 ± 0.14	3.37 ± 0.16	39.54 ± 4.97	22.67 ± 2.62	13.58 ± 0.6
L_6_	400	WW	42.81 ± 0.82	1.44 ± 0.1	40.16 ± 0.58	2.51 ± 0.16	44.3 ± 0.09	2.68 ± 0.11	30.46 ± 2.73	16.18 ± 0.87	16.63 ± 0.74
		D	42.23 ± 0.15	1.25 ± 0.08	40.95 ± 0.32	2.23 ± 0.03	44.16 ± 0.17	3.17 ± 0.09	34.5 ± 2.28	18.38 ± 0.19	13.98 ± 0.41
	800	WW	43.93 ± 0.52	1.09 ± 0.2	41.36 ± 0.66	3 ± 0.21	45.23 ± 0.11	3.4 ± 0.11	45.8 ± 8.09	14.13 ± 1.31	13.37 ± 0.4
		D	43.79 ± 0.62	1.14 ± 0.14	39.67 ± 0.84	2.28 ± 0.21	44.29 ± 1.13	3.8 ± 0.18	40.73 ± 4.67	17.95 ± 1.53	11.72 ± 0.35
*p*-Value of F-test			Pw = NS	Pw = NS	Pw < 0.001	Pw < 0.01	Pw = NS	Pw < 0.001	Pw < 0.01	Pw < 0.01	Pw < 0.001
			PCO_2_ < 0.001	PCO_2_ = NS	PCO_2_ = NS	PCO_2_ < 0.01	PCO_2_ < 0.001	PCO_2_ < 0.01	PCO_2_ = NS	PCO_2_ = NS	PCO_2_ < 0.01
			PG = NS	PG = NS	PG < 0.05	PG = NS	PG = NS	PG < 0.01	PG = NS	PG = NS	PG < 0.01
				Pw × G < 0.05		Pw × CO_2_ < 0.01				Pw × CO_2_ < 0.05	

WW is well-watered, D is drought stress, Pw is the *p*-Value derived from the F-test performed for the comparison water levels, PCO_2_ is the *p*-Value derived from the F-test performed for the comparison CO_2_ conditions, PG is the *p*-Value derived from the F-test performed for the comparison among genotypes, Pw × G is the *p*-Value derived from the F-test performed for the comparison of interaction between water levels and genotypes, Pw × CO_2_ is the *p*-Value derived from the F-test performed for the comparison of interaction between water levels and CO_2_ conditions, NS is non-significant, C is stem carbon (mg g^−1^), SN is stem nitrogen (mg g^−1^), LC is leaf carbon (mg g^−1^), LN is leaf nitrogen (mg g^−1^), GC is grain carbon (mg g^−1^), GN is grain nitrogen (mg g^−1^), SCN is stem carbon to nitrogen ratio, LCN is leaf carbon to nitrogen ratio, and GCN is grain carbon to nitrogen ratio.

## Data Availability

The original contributions presented in the study are included in the article/Supplementary Material; further inquiries can be directed to the corresponding authors.

## Supplementary Materials

The following supporting information can be downloaded at: https://www.mdpi.com/article/10.3390/plants13202942/s1, Table S1: Parentage, pedigree, and source of derivatives of bread wheat used in current experiment.; Table S2: Threshold value of FTSW (C_FTSW_) for 6 wheat genotypes under ambient and elevated CO_2_.
