# Supplementary material for: Drought Stress, Elevated CO2 and Their Combination Differentially Affect Carbon and Nitrogen in Different Organs of Six Spring Wheat Genotypes
Source: Plants (Basel). 2024 Oct 21;13(20):2942. doi: 10.3390/plants13202942 (PMC11511181; doi:10.3390/plants13202942)
Supplement: Supplementary file 1 [file plants-13-02942-s001.zip › plants-3248376-supplementary.pdf]

Table S1 Parentage, pedigree, and source of derivatives of bread wheat used in current experiment.

Genotypes	Parentage	Pedigree	Derivatives
L_1	H-1442/KACHU//BAJ #1	SDSS12B00859T-0Y-0B-0B-17Y-0B-0MXI	Landraces
L_2	QRO94.1.4/3/KACHU #1/KIRITATI//KACHU/4/PBW343*2/KUKUNA*2//FRTL/PIFED	SDSS12B01152T-0Y-0B-0B-4Y-0B-0MXI	Landraces
L_3	D67.2/PARANA 66.270//AE.SQUARROSA (185) /3/KACHU/4/BAJ #1	SDSS12B00840T-0Y-0B-0B-6Y-0B-0MXI	Synthetic bread wheat
L_4	BCN//SORA/AE.SQUARROSA (323)/4/ WBLL1/KUKUNA//TACUPETO F2001/3/BAJ #1/5/SERI.1B//KAUZ/HEVO/3/AMAD*2/4/KIRITATI	SDSS12B00908T-0Y-0B-0B-2Y-0B-0MXI	Synthetic bread wheat
L_5	Vorobey	CMSS96Y02555S	Advance line
L_6	AMSEL/ATTILA//INQ.91/PEW'S' (Punjab-11)	Pb.30196-1A-0A-2A-0A	Approved variety

Table S2 Threshold value of FTSW (C_{FTSW}) for 6 wheat genotypes under ambient and elevated CO_2.

**Genotype**	**L_1**	**L_2**	**L_3**	**L_4**	**L_5**	**L_6**
**C_{FTSW}** 400 ppm	0.61±0.13	0.46±0.03	0.39±0.02	0.61±0.08	0.55±0.04	0.43±0.05
**C_{FTSW}** 800 ppm	0.55±0.08	0.53±0.09	0.72±0.05	0.53±0.05	0.72±0.04	0.64±0.11
